# The Human Penile Fibro-Vascular Assembly Requires the Integrity of Ten Fibro-Ligaments

**DOI:** 10.3390/life15091492

**Published:** 2025-09-22

**Authors:** Heng-Shuen Chen, Chu-Wen Fang, Raymond W. M. Tsai, Chih-Yuan Hsu, Geng-Long Hsu, Hsiu-Chen Lu, Mang-Hung Tsai, Jeff S. C. Chueh

**Affiliations:** 1 Microsurgical Potency Reconstruction and Research Center, Puli Christian Hospital, No. 1, Tieshan Rd., Puli, Nantou 54546, Taiwan; chenhs@mail.pch.org.tw (H.-S.C.); raymondtsai@hotmail.com (R.W.M.T.); mitchaellhsu@gmail.com (C.-Y.H.); 2 Graduate Institute of Clinical Medicine, College of Medicine, National Taiwan University, Taipei 100225, Taiwan; 007abao@gmail.com (C.-W.F.); scchueh@ntu.edu.tw (J.S.C.C.); 3 Microsurgical Potency Reconstruction Center, Shu-Tien Urology Ophthalmology Clinic, The Yin Shu-Tien Medical Foundation, Taipei 10662, Taiwan; 4 Educational Resources Section, China Medical University Hospital, Taichung 40402, Taiwan; luhc.5512@gmail.com; 5 Department of Anatomy, China Medical University Hospital, Taichung 40402, Taiwan; mhtsai@mail.cmu.edu.tw

**Keywords:** arcuate pubic ligament, cavernosal ligament, distal ligament, fundiform ligament, spongiosal ligament, suspensory ligament, penile fibro-vascular assembly

## Abstract

Introduction: Penile fibro-ligaments have been extensively studied for many centuries; however, there is, unfortunately, a lack of thorough understanding. We aimed to bridge the research gap between anatomy and surgical reconstruction. Methods: We excluded cadaveric penises, already dissected and disassembled by medical students, that had damaged the corpora cavernosa (CC) or corpus spongiosum (CS). However, penises were included if both the ischiocavernosus and bulbospongiosus muscles were undamaged. A total of 8 out of 52 penises were meticulously examined. Our dissection findings were supplemented with 101 cadaveric photos, 255 penile vascular surgeries, 11 CT imaging scans, and 8 MRI imaging scans. The combined understanding was reprocessed with radiographic imaging, and patients underwent penile surgeries, notably eight hypospadias surgeries, and eight penile elongation surgeries were performed elsewhere. Results: Bilaterally, the penile CC is primarily anchored to the pelvic wall through the cavernosal ligament (CL = 2), while the CS is connected to the urinary sphincter. The suspensory, fundiform, and arcuate pubic ligament (two anatomically and one functionally) assist in stabilizing and holding the penile shaft to the pelvic wall. Distally, a distal ligament (DL = 1) and spongiosal ligament (SL = 1) extend the CC and CS to the glans penis and frenulum, ensuring urethral patency when necessary. The CC is encircled by a bi-layered tunica consisting of a 360° inner circular and a 300° outer longitudinal tunica. The ischiocavernosus muscle wraps around the penile crus and envelops the CL, connecting to the ischial tuberosity. The CS is partially surrounded by the bulbospongiosus muscle proximally and receives the SL distally. The entire penis interconnects with the skeletal muscle of the urogenital diaphragm. Conclusion: The physiological integrity of the human penis relies on ten anatomically and six functionally fibro-muscular ligaments.

## 1. Introduction

### Practitioner Points

1. As a male potency reconstructive practitioner, maintaining the integrity of the penile fibro-vascular assembly is second to none. The human penis is the ultimate coevolutionary nature as an intromittent organ. The penile fibro-vascular assembly relies on a combination to ensure its physiological integrity.
2. The penile fibrous, anatomical ten and functionally six, play a structural role in supporting and stabilizing the various components of the penis; these ligaments include the arcuate pubic ligament, cavernosal ligaments, distal ligament, fundiform ligament, spongiosal ligament, and suspensory ligament.
3. Given the importance of these ligaments in maintaining penile sexual health, caution should be taken when considering procedures such as ligamentolysis, which involves the release or modification of these ligaments; preserving the integrity and functionality of the ligaments is essential for ensuring optimal physiological and functional outcomes in the penile fibro-vascular assembly.

It is widely accepted that evolution plays a significant role in shaping the development of animal species on our planet [1]. Among these, humans are considered the most advanced and have dominated the Earth for three thousand centuries [2]. An intriguing aspect of human evolution is the coevolution of male genitalia, resulting in the unique intriguing micro-architectures of the penis. This unique structure enables piston-like mechanical interaction with the female vagina during copulation [3]. Such interaction is crucial for successful reproduction and the transmission of genetic material from one generation to the next, especially before the advent of in vitro fertilization (IVF) [4]. Recent reviews have provided substantial evidence for the evolution of genital structures, highlighting the coevolution of male and female genitalia in vertebrates [5,6]. Ongoing research endeavors to comprehend the mechanics of copulatory interaction, which necessitates a functional erectile system [7,8].

The human body is remarkable, equipped with a bony skull that safeguards the brain. This skull is connected to the body’s trunk through the cervical spine, which is essentially enhanced by fibrillar ligaments. The skeletal structure supports the body and enables unique movements, such as shaking the head, made possible by the atlas bone [9]. The body-supportive framework, formed by combining bony structures and fibro-muscular elements [10]. The body is articulated, with limbs serving the purposes of living and locomotion. The body core is centrally positioned on each level of the extremity, while the body elements are outside the body. The upper and lower limbs contain multiple joints at various levels, each associated with ten fingers and toes, as well as numerous ligaments [9].

Interestingly, the human penis is a fibrovascular assembly that has recently evolved as an independent compartment rather than an extension of the cardiovascular system [11]. It lacks bones and relies instead on centrally located hydraulic sinusoids to provide bony rigidity when needed [12]. As a protruding member of the extremity family, the human penis stands apart from others by lacking articulation and a central core supporter; however, it still adheres to the general design principle observed throughout the body, where a rigid supporter is typically centrally located in the case of the penis, this role is fulfilled by the functional sinusoids found in the CC and CS. Despite these differences, the penile design follows the same principle, albeit without the presence of bones. Therefore, the boneless penile CC can serve as an ideal milieu for applying Pascal’s law to the entire human body, given that the CC chamber remains free from veno-occlusive dysfunction (VOD) [13].

Furthermore, according to Pascal’s law, applied pressure remains constant within an enclosed space [14]. This implies that the penile structure requires collagen bundles, essential ingredients of bone, with the strength of steel to meet the requirements [15]. Not surprisingly, the CC can withstand intracorporeal pressure of 1800–2000 mmHg without rupturing [16]. To ensure this resilience, the outer shell of the CC, known as the bi-layered tunica albuginea (TA), particularly its outer coat, must be tightly stretched and exceptionally tough [17]. TA comprises multiple collagen bundles that run parallel to the skeletal structure, similar to the muscular system of the regular skeletal and muscular systems; categorically, it contains numerous fibrous ligaments.

A ligament is a fibrous connective tissue that connects bones or organs to other bones or organs [18]. As a result, ligaments are commonly found in various internal organs, including the fetal remnant, the peritoneal, and the falciform ligaments. Round ligaments are present in the urogenital system, where ligaments such as the inguinal, testicular, lacunar, fundiform, and suspensory ligaments are present [19,20]. Thus, the concept of the ligament is widely accepted in the field of penile fibro-vascular anatomy [21]. In our study, we explored the human penile fibro-vascular assembly using multiple-planar approaches; this exploration aimed to elucidate the last remaining independent vascular component in the human body [22]. However, due to the scarcity of detailed descriptions of the human penile fibro-ligaments in the medical literature, we were inspired to study the penile fibromuscular ligaments to understand their role in maintaining sexual health.

## 2. Materials and Methods

The Institute Review Board of China Medical University approved the study. The penises used in the study were already dissected, and the intromittent pendulous portion was disassembled into 2.5 cm cross-section blocks by medical students [23,24]. Penises were excluded from the study if both sides of the corpora cavernosa (CC) and corpus spongiosum (CS) were damaged or if a median sagittal resection had already been performed on the blocks. However, they were included in the study if a unilateral side of the CC remained undamaged with an intact Colle’s fascia. As a result, 15 out of 46 cadaveric penises were meticulously examined.

**Figure 1:**
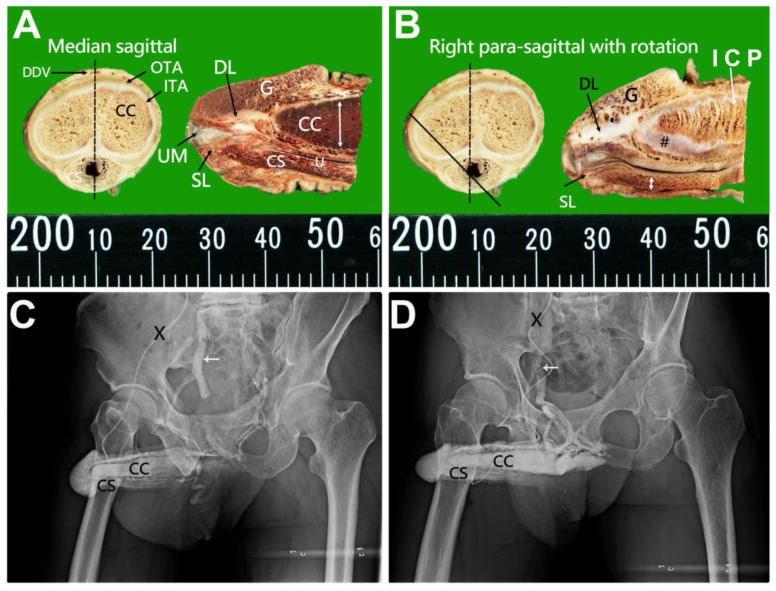
Photos of the distal penis and erection-related veins were demonstrated in cavernosograms. (**A**) A Median-sagittal section (black dotted line) of the human penis along the deep dorsal vein (DDV). It shows a bi-layered model of the outer tunica albuginea (OTA) and inner tunica albuginea (ITA), corpora cavernosa (CC) with distal ligament (DL) within the glans penis (G), corpus spongiosum (CS) housing the urethra (U) and spongiosal ligament (SL). Note that the urethral meatus and the tunica thickness are much thicker dorsally than the ventral portion (double-headed arrow). (**B**) A right para-sagittal section presents a similar scenario with DL, SL a gillnetting array, G, and intracavernosal pillars (ICP). Note the thickness of the spongiosal tunica (double-headed arrow) being identically thin, and a cavernosal membrane (Pound) is seen. (**C**) Cavernosogram was performed via a 19-gauge scalp needle (black cross) inserted in the corpora cavernosa (CC) communication to CS and G; subsequently, the contrast medium drains to the internal iliac vein (arrow). The drainage of blood is tremendous. (**D**) A similar scenario was demonstrated. Note that the primary drainage venous system is the right iliac vein (arrow).

**Figure 2:**
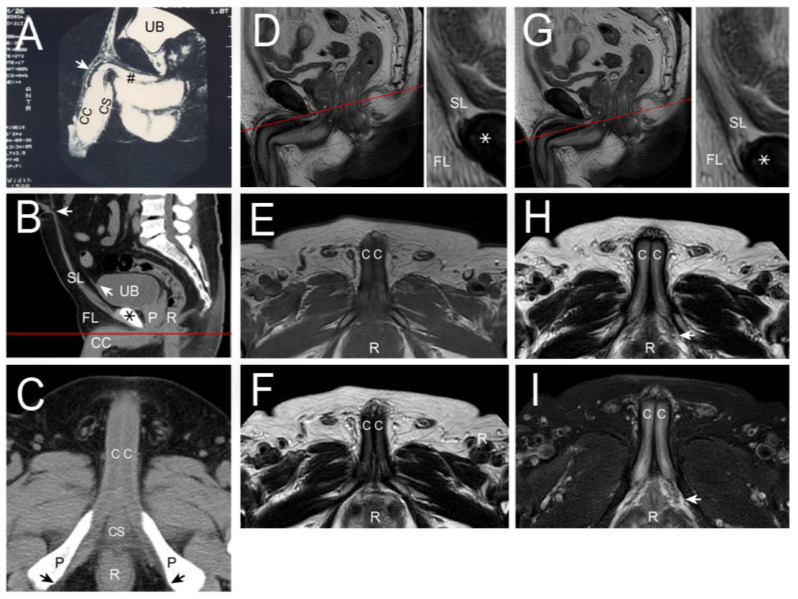
The human penile fibro-vascular assembly obtained from CT and MRI imaging. (**A**) In the CT imaging, a median sagittal section was made on a 28-year-old man with crural hypoplasia while the penile crus (pound) was petite, whose erection-related veins showed merely the proximal portion (arrow), otherwise masked by sinusoidal opacity. In addition, organoids were demonstrated, for the urinary bladder (UB), corpus cavernosum (CC), and corpus spongiosum (CS). (**B**) A median sagittal section of the pelvis was conducted to present the symphysis pubis (asterisk), the extension of the urinary bladder (UB) with the median umbilical ligament (oblique arrow), the prostate gland (P), the rectum (R), the suspensory ligament along the linear alba to the umbilicus (left-directed arrow). The section line, red color, was made below the symphysis pubis, resulting in the image of the following one. (**C**) Given the penis in erection status, it showed the penile corpora cavernosa (CC), (CS), the pubic bones (P), and the rectum (R) ischiocavernosus with cavernosal ligament socketed to the ischial tuberosity (arrow). (**D**) In MRI imaging, a cross-section was made below the symphysis pubis, red line, for comparison with CT imaging, again demonstrating the suspensory ligament (SL inserted right), fundiform ligament (FL, inserted right) and pubic bone (asterisk). For comparison, imaging was conducted in the T1 mode shown in (**E**) and T2 mode presented in (**F**) where the corpora cavernosa (CC) and the rectum (R) are well demonstrated, respectively. Note the right R points the right side. The erection-related veins could be well identified in the T2 mode. (**G**), however, not intriguing sufficiently. A cross-section was made below the symphysis pubis 2 mm lower, red line, and the mode was conducted in the T2 (**H**) and 2-FS (**I**), respectively. Note all the CC, R, FL, and SL mean the same. Overall, the T2 mode was preferred as its enhancing the fibrous tissue of the CC in this study.

**Figure 3:**
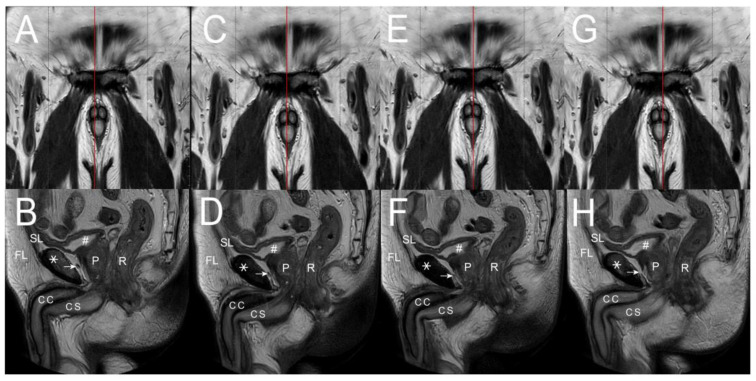
In the MRI T2 imaging study. (**A**) A 2 mm right para-sagittal section, red line, was made. (**B**) It demonstrated the symphysis pubis (asterisk), corpora cavernosa (CC), corpus spongiosum (CS), fundiform ligament (FL), suspensory ligament (SL), urinary bladder (pound), prostate gland (P), and rectum (R). Note the arcuate pubic ligament (arrow) scattered along the pubic bone (asterisk) to join the SL. Meanwhile, the erection-related veins, like the periprostatic ones, could be well seen. (**C**) The section was performed 1 mm medial to the film (**A**), resulting in the following imaging. (**D**) The deep dorsal vein climbs over the cavernosal vein along the corpora cavernosa (CC). A similar scenario was shown. (**E**) A median sagittal section, red line, was made to obtain similar methods in (**F**). (**G**) A 1 mm left para-sagittal section, red line, was further conducted to obtain the scenario of the above structures (**H**), which were unable to tell the bi-layered model of the tunica albuginea, DL, and SL identified in Figure 1A,B. Overall, the detailed three-dimensional architectures might be exclusively disclosed by the macroscopical dissection of the human cadaveric penises.

The distal blocks of the penises were prepared as median, 2.5 mm, and 5.0 mm sagittal sections from the median to the lateral. These sections were then examined microscopically, with analysis of the cut surfaces (Figure 1A,B). Additionally, clinical patients underwent hemodynamic cavernosograms by injecting 10 mL of contrast medium via a # 19 scalp needle to obtain intracavernous images (Figure 1C,D) [25]. Furthermore, dissection findings were correlated with 101 cadaveric photos and various diagnostic imaging techniques, including 255 sets of dual cavernosograpy from patients who underwent penile vascular surgery. Additionally, analysis was performed on 11 CT imaging scans (Ingenia 3T), 8 MRI (3T Philips MRI) imaging scans (Figure 2 and Figure 3), and five sets of spongiosographyy. Moreover, an information analysis was conducted on eight men with erectile dysfunction (ED) who had standard erectile capabilities before undergoing ligamentolysis elsewhere.

**Figure 4:**
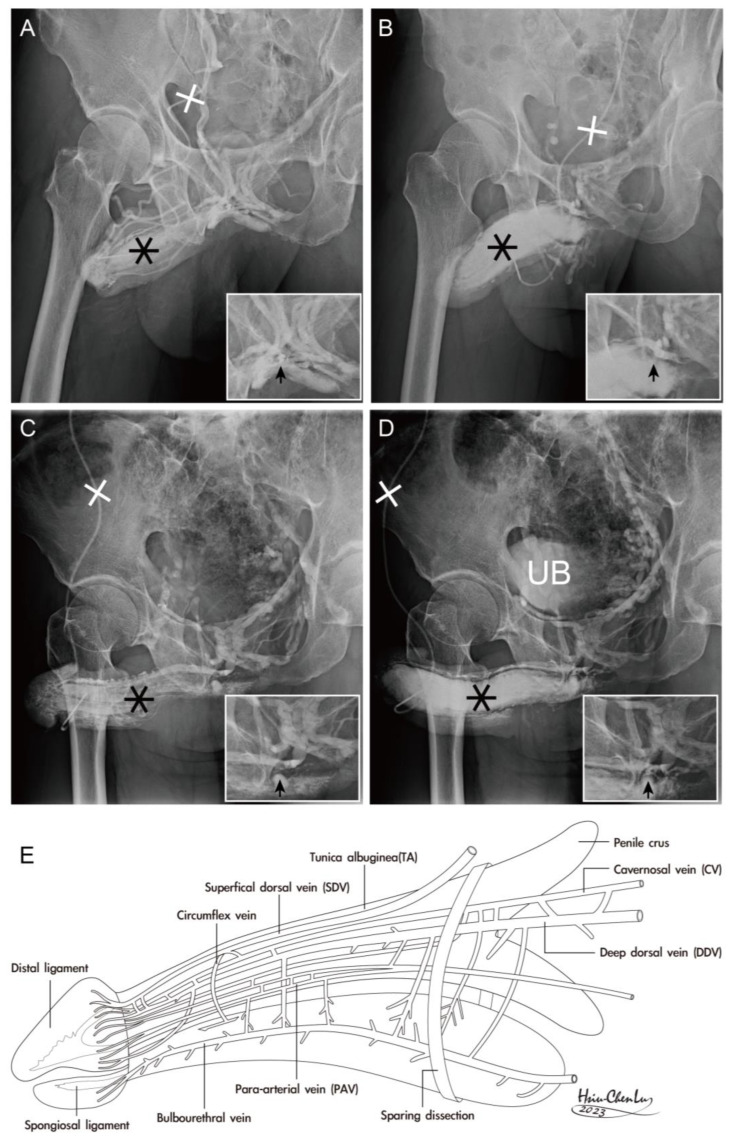
Physiologically, penile integrity is compromised after ligamentolysis for penile elongation elsewhere. (**A**) A 52-year-old patient sustained severe early detumescence and insufficient rigidity secondary to a penile ligamentolysis for penile lengthening elsewhere, even though his preoperative erection was gratifying. A cavernosogram is performed via a 19-gauge scalp needle (white cross) inserted into the corpora cavernosa (star)—subsequently, the contrast medium drains into the systemic circulation. The alignment integrity could not lash the erection-related veins (inserted, arrow). (**B**) A similar scenario was well demonstrated in a 47-year-old patient. (**C**) A 57-year-old patient sustained severe erectile dysfunction after two episodes of ligamentolysis for penile elongation internationally. The erection-related veins are distributed in a seaweed pattern. (**D**) These unlashed erection veins could not be limited after prostaglandin-E1 intracavernous injection, the erection rigidity is challenging to induce artificially. (**E**) An illustration was made to explain the lashing effect of the functional integrity by those coevolutionary fibromuscular ligaments (circle belt), such as the arcuate pubic, suspensory, and fundiform ligaments. Is the ligamentolysis, therefore, sustainable?

This analysis focused on dual cavernosography and emphasizing the venous vasculature related to erection (Figure 4). Additionally, eight patients with hypospadias were questioned to substantiate the role of the ejaculation bolus that might be related to the SL guarding meatal patency.

## 3. Results

Table 1 summarizes the ten human penile fibro-muscular ligaments. The human penis is a uniquely protruding organ composed of multiple fascial layers that surround the three cylinders of erectile sinusoids. It consists of the glans penis, the paired corpora cavernosa (CC) enveloped by the ischiocavernosus muscles, and the corpus spongiosum belted by the bulbospongiosus muscle. The CC is surrounded by a thin cavernosal membrane (Figure 1B). It is then anchored to the body trunk through the bilateral cavernosal ligament (CL = 2), while the CS contains the urethra connected to the urinary sphincter.

In the pubic region, proceeding from the median to lateral and from extra to intra-pelvis, there is the suspensory ligament (anatomically 2, functionally 1), the fundiform ligaments (anatomically 2, functionally 1), and the arcuate pubic ligament (anatomically 2, functionally 1), These ligaments sequentially stabilize and hold the penile shaft to the body trunk. Distally, there is a distal ligament (DL = 1), an os analog, and a spongiosal ligament (SL = 1). These ligaments project the CC and CS, respectively, to ensure the patency of the urethral meatus when necessary.

This physiological function is likely evidenced by the absence of hypospadias in the eight patients included in this study. Moreover, the SL is arrayed in a gillnetting manner with the primary 180° across-street position to DL (Figure 1A,B), which ensures the patency for ejaculate squirting. Finally, the CC is encircled by a bi-layered fibrous tunica model with 360° inner circular tunica albuginea and 300° outer longitudinal tunica albuginea, within which fibro-muscular tissue is expressed. The skeletal type includes bilateral CL, anchoring the penis to the pelvic wall at the ischial tuberosity. The CC sinusoids are 8.0–1.5 mm, evidenced in various imaging (Figure 1, Figure 2 and Figure 3) distally. Proximally, the bilateral ischiocavernosus sockets the CC, 300° outer longitudinal tunica, and DL in the glans penis, bulbospongiosus partially surrounds the CS and forms the ventral thickening of the outer tunica at the 5 and 7 O’clock positions, respectively, and a spongiosal ligament that anchors the CS to the frenulum. Proximally, the entire penis intertwines with the skeletal muscle of the urogenital diaphragm. The smooth component makes an exclusively hydraulic milieu, such as the penile artery, venous structure, intracavernosal pillars, and three specific sinusoids.

**Figure 5:**
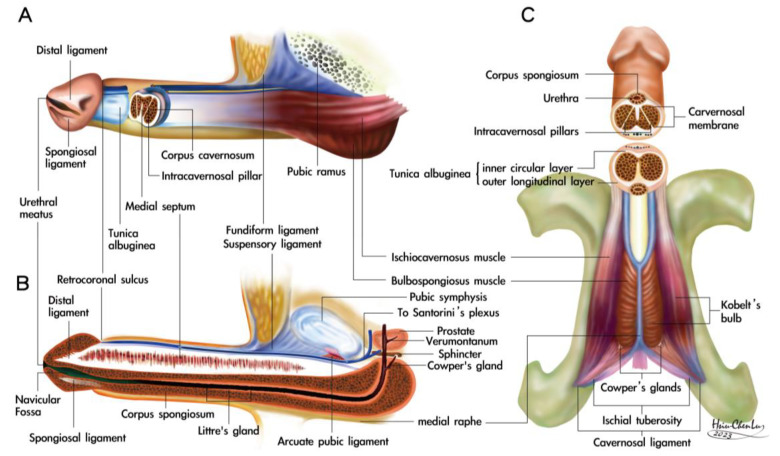
Schematic illustration of a three-dimensional view of the human penis. (**A**) Lateral aspect. The corpora cavernosa are held by the fundiform ligament from the pubic periosteum to the Colle’s fascia, adhered to the tunica albuginea. The glans penis caps it, mimicking a helmet for safety’s sake, and is buttered by a distal ligament. The bulbospongiosus muscle wraps the corpus spongiosum. The muscle fibers are primarily oblique, and distal threads are bundled, forming the thicker ventral thickening of the outer tunica at the 5 and 7 O’clock positions, respectively. The corpora cavernosa are surrounded by the tunica albuginea, a bi-layered model (an inner circular 360° and 300° outer longitudinal layer with multiple sublayers). The more distal and numerous intracavernosal pillars continue the inner circular layer. The corpus cavernosum is socketed in the ischiocavernosus muscle with the muscle fiber aligned in the longitudinal direction, scattered around 1 to 11 O’clock, respectively. (**B**) Medial aspect. The distal ligament is continued from the collagen bundles of the outer longitudinal layer of the tunica albuginea. It is an inelastic fibrous structure that supports the glans penis, mimicking a tree trunk of a Christmas tree. The incomplete septum is dorsally fenestrated. The corpus spongiosum contains the urethra. Note the fundiform ligament; the distal ligament is across-street with a spongiosal ligament. (**C**) Ventral aspect. The three-dimensional structure of the coevolutionary human penis is evident. The ischiocavernosus muscle is paired and sucked at the ischial tuberosity with the cavernosal ligament, then intertwined with the urogenital diaphragm. Each muscle boots its ipsilateral penile crus. Could you note the arrangement of the cavernosal ligament?

The sparing of either suspensory or fundiform ligament is challenging in penile ligamentolysis lengthening (Figure 4), as evidenced by the uneven lineation of the entire dorsal aspect of the CC and the loss of fibro-muscular banding that limits the erection-related veins. In addition, skeletal components encase the smooth part, which also serves as both a blood supply and drainage conduit in the paramount role of the integral hydraulic assembly. Consequently, a lateral aspect of the median-sagittal and para-medial sagittal section and a ventral view of the entire human penis is illustrated (Figure 5).

## 4. Discussion

In most quadriceps, the glans penis is supported by an os penis, which aids intromission and facilitates mating [26]. However, humans have lost the os penis, although there is an analog structure in histology [27]. Therefore, the human penis relies on the hydraulic CC to provide necessary rigidity during sexual activity. Thus, the ultimate hydraulic corpora cavernosa can meet the necessity of bony rigidity when required because a rigid intromittent human penis plays an essential role in intromission sex. Human penile erectile function is a delicately hydraulic event in which a seamless interplay of vascular, hormonal, psychological, neurological, pharmacological, sinusoidal, and metabolic contributors is involved [28]. The healthy vascular factor is essential in the sinusoidal function in the CC, CS, and the glans penis, independently. Physiologically, a penile, rigid erection ensues if and only if penile arterial, sinusoidal, and venous factors exist, while the corporal-veno-occlusive mechanism works vigorously. Thus, the integrity of penile fibro-vascular apparatus is a parallel prerequisite in erectile function corresponding with a cotton-soft flaccid arterial supply just being 2–3 mL/min, and bone-rigid erection the arterial supply being surged 30-fold to 60–80 mL/min for erection purposes [29], implying, upon activation, blood flow to the CC sinusoids will reach 240–320 mL/min if the penile arteries function bilaterally [30].

Many researchers are proud of the ultimate primordial human owning hydraulic erectile sinusoids gifted with magician-like erection rigidity for stroking the glans against the vaginal wall of a female counterpart. Otherwise, a penile bone is awkward in locomotion, for we erect human beings; therefore, humans must thank the creature for giving us a soft fibro-skeleton penis that can transform into a bony rigidity compartment, when necessary. There must be lots of coevolutionary fibrous ligaments inside and outside the penile fibro-vascular assembly, like a wooden house negating a nail. Whether the organ is healthy depends upon its ten ligamentous integrities. Interestingly, these ten numbers are inadvertently the same as the number of fingers and toes.

Although the human penis is generously extruded, its anatomy seems too simple-looking to conduct further study in the medical literature thus far [31]. The conventional human penile anatomy, a presumed illustration by sagacious Du Vinci in the medieval time, has been consistent for 550 years, albeit incorrectly, describing the tunica albuginea as a single circumferential layer with a uniform thickness as well as strength, and the glans penis exclusively comprises the sinusoid [32]. Also, there is just one deep dorsal vein sandwiched by a pair of dorsal arteries for erection-related drainage between the buck’s fascia and the tunica albuginea [33]. Without this strong DL, the glans would be too weak to bear the buckling pressure generated during coitus. And cannot interpret many physiological phenomena, including integral erection strength transmitting to the glans tip, meatal patency for ejaculation, Glans nodding secondary to the intracavernous pressure surge during ejaculation rhythm, and the pull-back force against the glans penis during anal constriction. The human penis parallels the structure of the human body, where the skeletal fibro-muscular wall encompasses those visceral organs, mainly composed of smooth muscles [34]. It is a boneless organ suspended from the prepubic region in front and strongly socketing to the pubic ramus via the tenacious CL at the ischial tuberosity. The organ leans upon the suspensory ligament, an extension of the linea alba. An erect penis is analogous to an athletic diver without upper extremities standing on a springboard, ready to dive. Thus, the glans penis corresponds to the head, the penile shaft corresponds to the body trunk, and the penile crura correspond to the legs.

The de novo penile fibro-vascular assembly involves the presence of the bi-layered tunica albuginea (tA), which plays a crucial role in the erection process [35]. The TA comprises an inner circular layer (360°) and an outer longitudinal coat (300°), serving as the fundamental structure for penile morphology and protecting the penile prosthesis [36]. Despite the significant volume difference between the body and the penis, the principle of having a fibro-muscular outer wall encasing the smooth component remains the same [37]. This implies that functional compartments composed of smooth muscles require protection from a more robust skeletal fibro-muscular outer shell. The inspiration for relevant research and studies often comes from patients’ inquiries during daily medical practice and virtual anatomical dissections, which reveal newly discovered penile venous anatomy concerning the penile fibroskeleton [38]. An analogy can be drawn to how an umbrella protects against rain, as a stick (representing the distal ligament) is necessary to function effectively.

While advanced technologies, such as MRI and artificial intelligence, have revolutionized medical research [39], the contribution of cadaveric dissection remains rudimentary but essential. Some researchers argue that the most advanced imaging methods, like CT-penile imaging [40] and MRI-penile imaging [41], are the best tools for demonstrating the penile fibro-vascular assembly. However, it is intriguing that specific critical penile structures remained elusive until recent research efforts, which were inspired by patient questions and involved multiplanar collaborations, including traditional cadaveric dissection and conventional cavernosography. Traditional dissection remains the primary method for revealing the intricate lattice array of the spongiosal ligament (SL), which is often scarce in patients with hypospadias. Combining the distal ligament, mimicking two poles, is crucial in ensuring urethral meatus potency (Figure 1).

Similarly, the conventional anatomy dissection method is indispensable for penile erection-related venous vasculature. This study combines various techniques, including cadaveric dissection, X-ray imaging, CT scanning, and MRI imaging, to comprehensively explore different aspects of penile anatomy. While innovation is important, it can be done without completely abandoning conventional methods in favor of the most advanced facilities. Traditional and advanced approaches have unique contributions, and a virtual anatomic dissection is an invaluable tool for understanding complex anatomical structures. Ultimately, the truth and value of medical practice are determined by practical application and patient outcomes.

CT and MRI can help visualize the deep-seated fibrous ligaments in the penile anatomy [42]. These ligaments, including the suspensory ligament, fundiform ligament, and arcuate pubic ligament, support the penis in the prepubic region. While advanced imaging technology allows researchers to observe these ligaments more efficiently, they can be challenging to identify during penile lengthening procedures such as ligamentolysis [43,44]. Those ligament lysis procedures are not considered viable in modern expertise. Researchers have encountered difficulties in preserving the banding ability of fibro-ligaments during these procedures, leading to compromised support for erection-related veins and subsequent veno-occlusive dysfunction (VOD). However, the sample size is small (eight cases), and severe VOD was observed in this series of patients who underwent penile lengthening. In male potency reconstruction, it is crucial to recognize the integral relationship between the architecture of skeletal and smooth muscles. This understanding extends to various approaches for penile erection restoration and morphology reconstruction [45], emphasizing the importance of considering both structural and functional aspects of the penis [46].

## 5. Conclusions

Maintaining the integrity of the penile fibro-vascular assembly is crucial due to its coevolutionary nature as an intromittent organ. The penile fibro-vascular assembly relies on a combination to ensure its physiological integrity. The anatomical ligaments play a structural role in supporting and stabilizing the various components of the penis; these ligaments include the cavernosal ligaments, suspensory ligament, fundiform ligament, arcuate pubic ligament, distal ligament, and spongiosal ligament. Given the importance of these ligaments in maintaining penile health and function, caution should indeed be exercised when considering procedures such as ligamentolysis, which involve the release or modification of these ligaments. Preserving the integrity and functionality of the ligaments is crucial for ensuring optimal physiological and functional outcomes in the penile fibro-vascular assembly.

## Figures and Tables

**Table 1:** Ten skeletal fibrous ligaments in the human penis based on de novo penile fibro-vascular assembly.

No	Terminology	Origin	Insertion	Number of Ligaments		Connecting Structures
	Ligament/T Ligament/Tendon			Anatomical Functional		
1	Cavernosal	Ischial tuberosity	Dorsal aspect of outer Tunica	2	1	Ischium and corpora cavernosa
2	Suspensory	Buck’s fascia	Pubic symphysis	2	1	Buck’s fascia∼Pubic symphysis
3	Fundiform	Lateral pubic bone	Lateral pubic bone	2	1	Lateral pubic bone∼Penile base
4	Arcuate pubic ligament Distal (os analog)	Buck’s fascia Corpora cavernosa	Medial surface of pubic ramus Glans penis	2 1	1 1	Glans penis and corpora cavernosa Gans penis and corpora cavernosa
5	Spongiosal	Corpus spongiosum	Frenulum	1	1	Frenulum and corpus spongiosum
Total				10	6	

## Data Availability

The original contributions presented in this study are included in the article. Further inquiries can be directed to the corresponding author.
