# Correction: Chen et al. The Human Penile Fibro-Vascular Assembly Requires the Integrity of Ten Fibro-Ligaments. *Life* 2025, *15*, 1492

**DOI:** 10.3390/life16040543

**Published:** 2026-03-25

**Authors:** Heng-Shuen Chen, Chu-Wen Fang, Raymond W. M. Tsai, Chih-Yuan Hsu, Geng-Long Hsu, Hsiu-Chen Lu, Mang-Hung Tsai, Jeff S. C. Chueh

**Affiliations:** 1 Microsurgical Potency Reconstruction and Research Center, Puli Christian Hospital, No. 1, Tieshan Rd., Puli, Nantou 54546, Taiwan; chenhs@mail.pch.org.tw (H.-S.C.); raymondtsai@hotmail.com (R.W.M.T.); mitchaellhsu@gmail.com (C.-Y.H.); 2 Graduate Institute of Clinical Medicine, College of Medicine, National Taiwan University, Taipei 100225, Taiwan; 007abao@gmail.com (C.-W.F.); scchueh@ntu.edu.tw (J.S.C.C.); 3 Division of Urology, Department of Surgery, Changhua Christian Hospital, Changhua 50006, Taiwan; 4 Microsurgical Potency Reconstruction Center, Shu-Tien Urology Ophthalmology Clinic, The Yin Shu-Tien Medical Foundation, Taipei 10662, Taiwan; 5 Educational Resources Section, China Medical University Hospital, Taichung 40402, Taiwan; luhc.5512@gmail.com; 6 Department of Anatomy, China Medical University Hospital, Taichung 40402, Taiwan; mhtsai@mail.cmu.edu.tw

## Additional Affiliation(s)

In the published publication [1], there was an error regarding the affiliation for Chu-Wen Fang. In addition to affiliation 2, the updated affiliation should include affiliation 3 as follows:

3. Division of Urology, Department of Surgery, Changhua Christian Hospital, Changhua 50006, Taiwan

The authors state that the scientific conclusions are unaffected. This correction was approved by the Academic Editor. The original publication has also been updated.
